# The Role of the Nurse Coordinator in Spina Bifida Clinics

**DOI:** 10.1100/tsw.2007.305

**Published:** 2007-11-26

**Authors:** Mary Jo Dunleavy

**Affiliations:** Department of Surgery, Children's Hospital Boston, 300 Longwood Avenue, Boston, MA 02115, USA

**Keywords:** nurse coordinator, role of nurse coordinator, spina bifida clinics, spina bifida

## Abstract

There are numerous multidisciplinary spina bifida (SB) clinics (typically including urology, orthopedics, neurosurgery, developmental pediatrics, physiatry, nursing, social work, and physical and occupational therapy) throughout the U.S. Many SB clinics have a nurse coordinator. The coordinator's role is truly multifaceted. It goes far beyond coordinating the clinic visit in which patients and families are seen for care. The frequency of clinical visits varies from program to program, from a few hours once a month to a full day every week. This role encompasses many aspects of care for this complex patient population, which will be described.

## INTRODUCTION

Spina bifida (SB) is one of the most complex congenital disorders, affecting multiple body systems. It is among the most common birth defects that cause permanent disability[1,2]. According to the National Center for Health Statistics, the rate in 2004 was 10.39 per 100,000 live births[3]. SB can cause sensory, motor, autonomic, cognitive, orthopedic, bladder, bowel, and sexual dysfunction throughout an individual's life[4].

To achieve optimal function, coordination of care for this patient population is extremely important and highly beneficial to the patient and family. The Spina Bifida Association[5] supports outcomes of care that include provision of "ongoing, comprehensive, coordinated care throughout the life span"[6]. The nurse coordinator is an integral player in achieving this goal. It includes facilitating the health care providers during clinic, as well as the patients and families. Likewise, it includes coordinating future clinic visits, with appropriate studies and referrals as recommended by the team.

## PRENATAL COUNSELING

The nurse must have an in-depth understanding of the complexity of needs of all patients with SB. As advances in health care and technology have made prenatal diagnosis of SB more commonplace, care throughout the life span often begins in the prenatal stage. The nurse coordinator is often part of a multidisciplinary team of professionals that is involved in counseling and educating expectant parents and their family about SB. Providing accurate and realistic information to families is a priority. Awareness of SB resources, both locally and nationally, is important in order to provide the necessary guidance and support to these families. Parents of other children with SB can be an invaluable support to the pregnant woman and her family at this point. The coordinator is often the facilitator of such connections. The nurse has the opportunity to develop and foster therapeutic, supportive relationships with the parents of children with SB during this prenatal period that will continue for many years to come.

## ROLE AS EDUCATOR

After the baby is born, the nurse coordinator is often the filter through which information is provided to the parents. S/he can help to interpret information given by the neurosurgeon and other providers that may seem daunting to the new parents. In addition to the dynamics and stress of parenthood, families face the challenge of caring for a baby with a disability. Aside from the "usual" newborn care, parents may need to learn, among other things, signs and symptoms of shunt malfunction, care of the surgical wound, or how to catheterize their baby. The role of educator is inherent in that of a nurse, but with a chronic, disabling condition, this becomes absolutely crucial. Each developmental stage will bring about new challenges. Anticipatory guidance is key to help the child and family to set and achieve realistic goals that will help the child to become an independent adult eventually.

## COORDINATOR OF CARE

One of the important aspects of this role is to coordinate the clinic session in which the patient is seen for care. Prior to each clinic, the nurse must prepare by reviewing records, establishing the purpose and goal of each visit, and ensuring that all necessary studies are completed or scheduled for that visit. Consultation with other health team members occurs, often in advance, to help develop an appropriate plan of care. During the clinic visit, the nurse coordinator may provide direct patient care and education, collaborate with and facilitate the various multidisciplinary team members, facilitate patient care visits, and may even act as a "traffic cop" to help maintain seamless patient flow.

The job does not end after the clinic day is complete, however. The follow-up plan that is needed to maintain optimal care is ongoing. Not only does the nurse coordinator obtain and review recommendations from each health care team member after each clinic visit and implement those plans, the nurse also helps to communicate such plans to the patient, family, and others involved in the patient's care, including the child's primary physician, Early Intervention Programs, schools, etc. Collaboration among members of the health care team is essential. Follow-up care can include reviewing results of various tests, such as laboratory, imaging, and urodynamics; implementing treatment plans for urinary tract infections and constipation; and completing phone calls to parents, pediatricians, and other providers. All of these activities require documentation in the patient's medical record. Coordinating follow-up also involves making parents aware of resources and support networks for families on the local, regional, and national level.

Coordination requires a fund of knowledge regarding insurance coverage for medical supplies, such as catheters and braces, as well as helping families to access this coverage by supplying letters of medical necessity and prescriptions.

The nurse coordinator may also be involved in planning and facilitating educational sessions for patients and families, or planning workshops and/or conferences for other health care professionals. Many nurse coordinators are active members of their state or regional SB Association as well as the national SB Association.

## TRIAGE ROLE

The nurse coordinator is often the primary contact person for patients with SB and their families to help with triage issues and concerns that arise during and between clinic sessions. Phone triage is a large part of this role. There are several life-threatening situations that can present themselves in this patient population, such as shunt malfunction, bladder rupture in patients who have had a bladder augmentation, or severe stridor or breathing problems secondary to Chiari malformation. There are also less urgent, but still concerning, situations, such as skin breakdown, fractures, problems with bowel management, urinary tract infections, or signs of spinal cord tethering. Information may come directly from parents or patients, or it may come from primary care or other health care providers. The nurse coordinator is often the "point person" for triaging these situations. S/he must be able to synthesize, interpret, and prioritize information presented to ensure that the child is accessing appropriate and safe care in a timely fashion.

## NEUROSURGICAL ISSUES

There are several neurosurgical issues that need to be monitored, regardless of age, in the SB population. Approximately 80% of patients with SB require shunting for hydrocephalus[7]. Understanding the overt as well as subtle signs and symptoms of shunt malfunction is extremely important as they can be subtle[4]. Chiari type II malformation (displacement of cerebellar tonsils downward into the cervical spine) is present in the majority of patients with SB, but only about 33% of patients become symptomatic[8]. Symptoms may vary, depending on the age of presentation (in newborns/infants: stridor, difficulty swallowing, weak cry; while in older children: upper extremity spasticity, poor balance)[8]. However, it is the leading cause of death, especially in younger children[8,9]. The nurse coordinator plays an important role, not only in educating patients and families about these conditions, but also in the ability to assess for associated symptoms, whether overt or subtle.

## BOWEL AND BLADDER MANAGEMENT

Bowel and bladder management in children with SB can require a great deal of time and patience on both the parent/patient's part and the care provider. Learning to catheterize a child is a significant undertaking for a parent. The nurse coordinator is usually the one who teaches this to a parent, and, as the child gets older, to the child him/herself. There may be ongoing issues related to catheterization, medications, infections, and incontinence that require frequent "tweaking" that is managed by the nurse in collaboration with the urologist.

Bowel management in children with SB is a lifelong challenge and should begin in the newborn period to prevent chronic constipation. As the child develops, strategies to initiate a bowel program to minimize incontinence should occur[4,6]. The nurse coordinator is most often the contact person in managing the child's bowel program. This may be done in collaboration with other health team members, but s/he is usually the one to implement the plan. This involves ongoing assessment, either by phone or in person, and requires vigilant follow through.

## ORTHOPEDIC ISSUES

Mobility is often affected in patients with SB. Deformities of the lower extremities may occur, including club feet and other foot/ankle deformities, dislocated hips, and joint contractures. Spinal deformities may include scoliosis and kyphosis. Pathological fractures associated with osteoporosis may occur. These may be long undetected or even easily attributed to abuse by a clinician unfamiliar with SB. The nurse coordinator can help both patients and families, as well as others involved in the care of a person with SB, to understand how this occurs as well as implementing preventive measures. Some of the goals of orthopedic care include maximizing function, maximizing mobility, and maintaining and protecting skin integrity[6,9]. This may require surgical intervention. Collaboration with the orthopedist and physical therapist is important in order to provide proper education to the patient and family.

## SEXUALITY ISSUES

It is important to begin providing information to parents about a child's expected sexual function early in the child's life. Parents often want to know this information even though they may not voice their concerns. It will help them later on when having frank discussions about sexuality with their child[6]. The possibility of precocious puberty, which occurs in about 15% of girls with SB, should be discussed with parents of affected females[6]. Referral to an endocrinologist may be indicated for possible hormonal suppression to delay this early onset of puberty[4,6,9].

Education about sexual function should be provided in the pre- and early teen years to both sexes. It may be helpful to have a health care provider of the same sex provide that information to the teen. This can be done by various health care team members, but most often by the nurse coordinator, urologist, or pediatrician. As with any teenager, patients with SB should be taught about protecting against sexually transmitted diseases and sexual abuse, as well as birth control. Erectile and ejaculatory function will vary in a male with SB, depending on the level of the lesion. Females with SB should know that their fertility is near normal. Both sexes should understand that practicing good hygiene, and maintaining good bowel and bladder programs play important roles in developing healthy, happy relationships. The nurse coordinator can provide ongoing assessment, education, and guidance for the often-sensitive issues related to sexuality.

## FOLIC ACID

All women of child-bearing age should take 400 µg folic acid (found in a multivitamin) to prevent the birth of a child with a neural tube defect[1,3,10]. However, a woman with SB has an increased risk and should take 4 mg (4000 µg) of folic acid daily. This amount requires a prescription. It is important that the nurse coordinator understands the role of folic acid in preventing neural tube defects because they are often the one who will educate the parents of a child with SB regarding future pregnancies, as well as the girl with SB as she gets older.

## LATEX ALLERGY

There is a high prevalence (between 30 and 72%)[9] of latex allergy in individuals with SB. Nurse coordinators need to educate patients, families, and other clinicians about this, both in the hospital/clinic setting and the outside general community. They have been instrumental in implementing changes to avoid continued exposure to latex, which can lead to life-threatening, allergic reactions[11].

## SKIN CARE ISSUES

Due to insensate skin, patients with SB are at high risk for skin breakdown throughout their life. Education is key to prevention. The nurse coordinator is instrumental in providing education to parents in the newborn period, and then teaching the child to learn skills in self-inspection as s/he develops. The parent AND child need to learn common causes of breakdown, such as prolonged sitting, incontinence, ill-fitting shoes and/or braces, as well as preventive measures to avoid such problems. Despite this, skin breakdown does occur. The nurse is in a central position to help manage and coordinate treatment. This may be done in collaboration with other experts, such as wound and ostomy care nurses, and plastic surgeons.

## PSYCHOSOCIAL ISSUES

Learning disabilities are common among those with SB. Specific problem areas include attention and executive functioning, perceptual motor issues, comprehension, and problem solving or decision making. Such challenges can obviously affect many aspects of life including academics, learning self-care (such as catheterization), social competence, and functioning in the workplace[9,12]. It is essential for the nurse coordinator to have an understanding of this when providing care and education to the patient with SB as it will affect every aspect of that person's life. Neuropsychological testing can help to identify strengths and weaknesses, which can be useful to parents and teachers, but also to the health care provider in teaching self-care and activity of daily living skills[12].

## ROLE AS RESOURCE

The nurse coordinator is often seen as a resource to many, inside of and away from the employing institution. Again, that role often begins in the prenatal period. It may include consultation with obstetricians and genetic counselors. Once the child is born, the nurse coordinator may collaborate with the neonatology and neurosurgery teams and help to coordinate discharge plans and teaching. Pediatricians are an important link in the care of children with SB. Oftentimes, a pediatrician may not have taken care of a newborn with SB, so the nurse coordinator is the person on whom they rely for sharing medical information and education on important issues. Early Intervention Programs, day care programs, and schools also look to the nurse coordinator for medical information and a better understanding of the child's condition and priority needs. The nurse is often involved in helping parents and schools to develop Individual Education Plans (IEP) that are appropriate for that child and that will meet all his/her needs. It is not uncommon to have a nurse coordinator attend IEP meetings or go to a school to teach the staff about SB.

Many SB clinics are held in teaching institutions across the country. The nurse coordinator is often a resource to medical students, interns, residents, fellows, and other nursing and clinical staff.

Children with SB may have frequent hospitalizations, whether for a surgical procedure or medical condition. The nurse coordinator can play a key role in ensuring that the hospitalization goes well. S/he is often the one with the most knowledge about a family situation or how that family deals with stress.

## CONCLUSION

There are many disciplines involved in the care of a person with SB. However, the nurse coordinator is in a position to provide a holistic approach to that care and can play a pivotal part in providing the "comprehensive, coordinated care" that the SB Association seeks for patients and families.

## ACKNOWLEDGEMENT

I am grateful to my colleagues, Stuart Bauer, MD, Nedda Hobbs, MD, Pixie Plummer, MD, Rosella Micalizzi, PNP, Ellen O'Donnell, PNP, and many members of the Nursing and Healthcare Professional Council of the Spina Bifida Association for their support and guidance in writing this article.
